# Grapevine Wastes: A Rich Source of Antioxidants and Other Biologically Active Compounds

**DOI:** 10.3390/antiox11020393

**Published:** 2022-02-15

**Authors:** Anda Maria Baroi, Mircea Popitiu, Irina Fierascu, Ionela-Daniela Sărdărescu, Radu Claudiu Fierascu

**Affiliations:** 1 National Institute for Research & Development in Chemistry and Petrochemistry—ICECHIM, 060021 Bucharest, Romania; anda.baroi@icechim.ro (A.M.B.); fierascu.radu@icechim.ro (R.C.F.); 2 Faculty of Horticulture, University of Agronomic Sciences and Veterinary Medicine of Bucharest, 011464 Bucharest, Romania; 3 Department of Vascular Surgery and Reconstructive Microsurgery, Victor Babes University of Medicine and Pharmacy, 300041 Timisoara, Romania; 4 National Research and Development Institute for Biotechnology in Horticulture, 117715 Stefanesti, Romania; ionela.toma93@yahoo.com; 5 Department of Science and Engineering of Oxide Materials and Nanomaterials, University “Politehnica” of Bucharest, 011061 Bucharest, Romania

**Keywords:** grapevine wastes, biological applications, antioxidant, viticulture

## Abstract

Wine production is one of the most critical agro-industrial sectors worldwide, generating large amounts of waste with negative environmental impacts, but also with high economic value and several potential applications. From wine shoots to grape pomace or seeds, all of the wastes are rich sources of bioactive compounds with beneficial effects for human health, with these compounds being raw materials for other industries such as the pharmaceutical, cosmetic or food industries. Furthermore, these compounds present health benefits such as being antioxidants, supporting the immune system, anti-tumoral, or preventing cardiovascular and neural diseases. The present work aims to be a critical discussion of the extraction methods used for bioactive compounds from grapevine waste and their beneficial effects on human health.

## 1. Introduction

**Figure 1:**
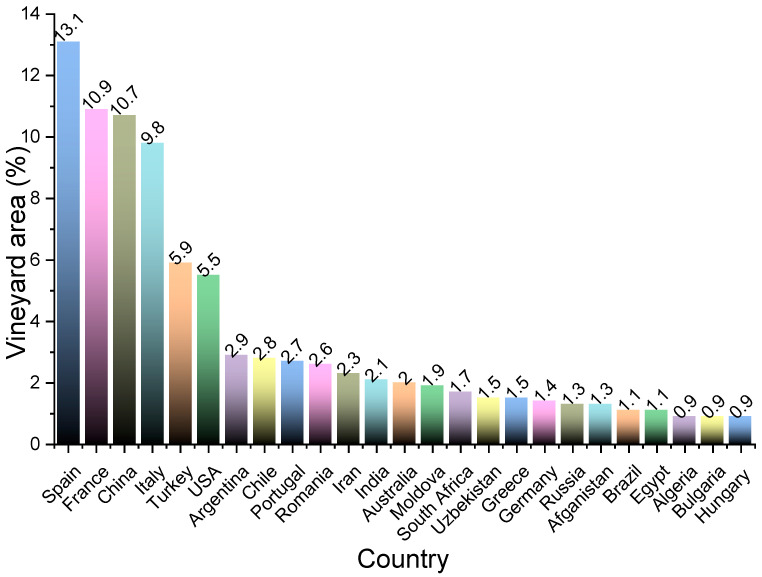
Percentages of vineyard areas of the main vine-growing countries in 2020.

Viticulture is one of the world’s most recognizable agricultural activities, with a global grape production of 77.8 million tons per year [1]. Interest in vineyards has grown significantly worldwide. In the last 5 years, the European Union (EU) vineyard has presented a stable balance between deforestation and the planting of new vineyards, while China attains 3rd place worldwide after Spain and France in vineyard area [2] (Figure 1, according to statistics provided by the International Organization of Vine and Wine).

**Figure 2:**
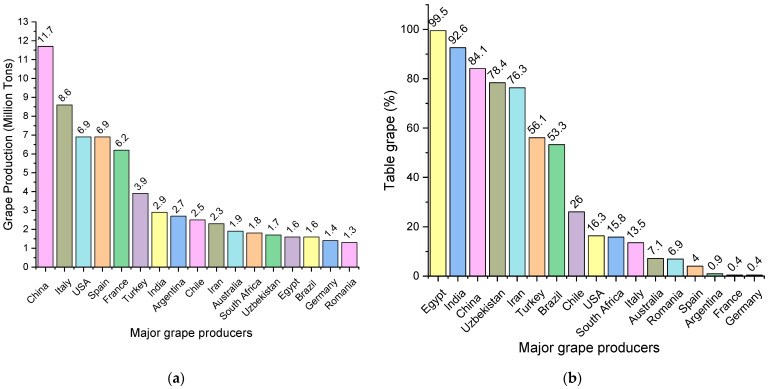

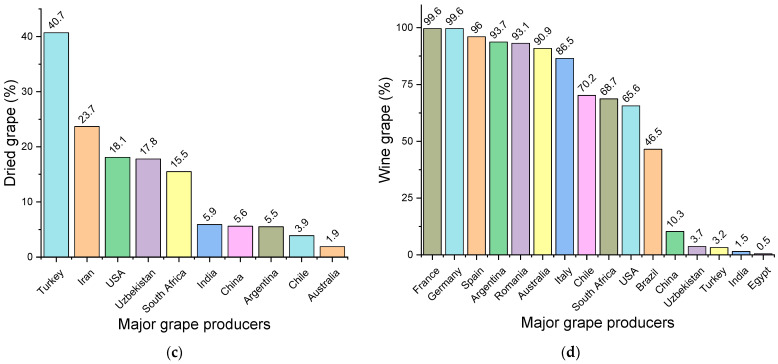
Grape production of the main producers and production per category (%): (**a**) grape production by country; (**b**) table grape production (%); (**c**) dried grape production (%); (**d**) wine grape production (%).

Grape production is divided into three main categories: wine grapes (57%), table grapes (36%) and dried grapes (7%) [2] (Figure 2, according to statistics provided by the International Organization of Vine and Wine).

Waste products are generated at each stage of the winemaking process [3]. There are two distinct categories that show the origin of winery wastes: those that result from the collection of grapes (solid wastes), and those that result from the winemaking process (liquid wastes) [4]. The first category broadly involves grape stalks (7.5% of total solid wastes generated by winery), grape pomace (45%), grape seeds (6%) [5,6], stems, as well as wine yeasts [7]. Pomace represents 20–25% of the initial weight of the grapes, being a solid residue resulting from the processes of fermentation and pressing of the grapes [8].

Wine yeast accounts for about 5% of the total weight of grapes, being rich in ethanol, tartaric acid, phenolic compounds and yeast cells [9]. Grape skins represent 7% of the total weight of the grapes, usually being removed before the fermentation stage, in order to avoid excessive astringency of the wine. They are a rich source of phenolic compounds (especially tannins, flavan-3-ols, hydroxycinnamic acids, monomeric and oligomeric flavonols and stilbenes) [10] and lignocellulosic compounds (hemicellulose, cellulose and lignin), most of them with antioxidant properties [7].

Grape seeds are rich in antioxidant compounds such as vitamin E, phenolic compounds, phytosterols, fibers, proteins, carbohydrates and minerals, especially lipids, and melatonin [11]. In contrast, grape skins and pulp are a rich source of fiber, phenolic acids (gallic acid, vanilla and caftaric acid), flavonols (quercetin, myricetin and kaempferol), and anthocyanins [12]. However, the wine industry produces, in a short period of time, a large amount of waste and by-products, representing about 30% of the initial weight of the grapes [13,14]. The accumulation of these wastes can cause economic and environmental issues due to the organic matter, acidic pH, salinity and heavy metal content [15].

In order to obtain the best-quality grapes, different management techniques were applied to vineyards, which involve cutting, irrigating and fertilizing [16]. Pruning plays an important role in the quality development of the vine and implicitly in the production of grapes, being performed in both cold and warm seasons. The pruning practice of vineyards in winter is the main source of vine residues, consisting of the accumulation of large amounts of vine shoots and canes [17]. The growing demand for grapes has automatically led to an impressive increase in both the area and the value of the vineyards. Thus, the accumulation of organic and inorganic waste causes major problems related to their economic and ecological management [18]. Among the wastes from the pruning of vines are also those from leaf trimming and cluster thinning namely stalks, unripe grapes, vine shoots, and leaves [18]. Crops grown for table grapes produce much more viticultural waste, because the growth of these crops is based primarily on improved quality and limited production [19,20]. The accumulation of viticultural waste has a negative impact both on the environment and human health through water pollution, oxygen consumption in the soil and groundwater, as well as through the attraction of vectors of disease spread [21]. Globally, there is a growing demand for healthy, natural food ingredients that can replace synthetic antioxidants and food preservatives [22]. Several scientific studies confirm the presence of phytochemicals (antioxidants) in grapevine and its by-products, as well as their potential applicability as (natural) added value in the food, pharmaceutical and cosmetic industries due to their nutritional benefits (dietary fiber, phenols, proteins and lipids). They can also be used as fertilizers for soils or as biomass for energy production [9,23]. Vine leaves constitute important sources of organic acids, lipids and polyphenols, having applicability in the cosmetic industry [15]

Furthermore, vineyards waste together with pomace may also be used as: antimicrobial and antioxidant agents; natural additives, improving the nutritional quality of food; fillers in food packaging formulas [24]; or as a hardening agent [25]. The recovery levels of bioactive compounds are closely linked to the vinification procedures as well as to the grape variety considered [26].

The present work aims to be a critical discussion of the extraction methods of bioactive compounds from grapevine waste and their beneficial effects on human health.

The methodology used in the present review involved the interrogation of available databases (Web of Science, Scopus, SpringerLink, and Google Scholar) for studies involving grapevine wastes (composition, separation, application in different industries) using as primary search keywords “grapevine”, “wastes”, “composition”, respectively the targeted industries. Following the primary search, the results were filtered by reading the abstracts of the articles, in order to remove false positive results. In the next step, all selected articles considered eligible were retrieved and independently assessed, considering their content, publication year and relevance for the present review. From the final selection we excluded reviews, protocols, theoretical papers, editorials, letters to the Editor, and book reviews. The selected articles were evaluated by the authors and relevant information was collected in order to be used in the review article.

## 2. Extraction Methods of Bioactive Compounds from Grapevine Waste

**Figure 3:**
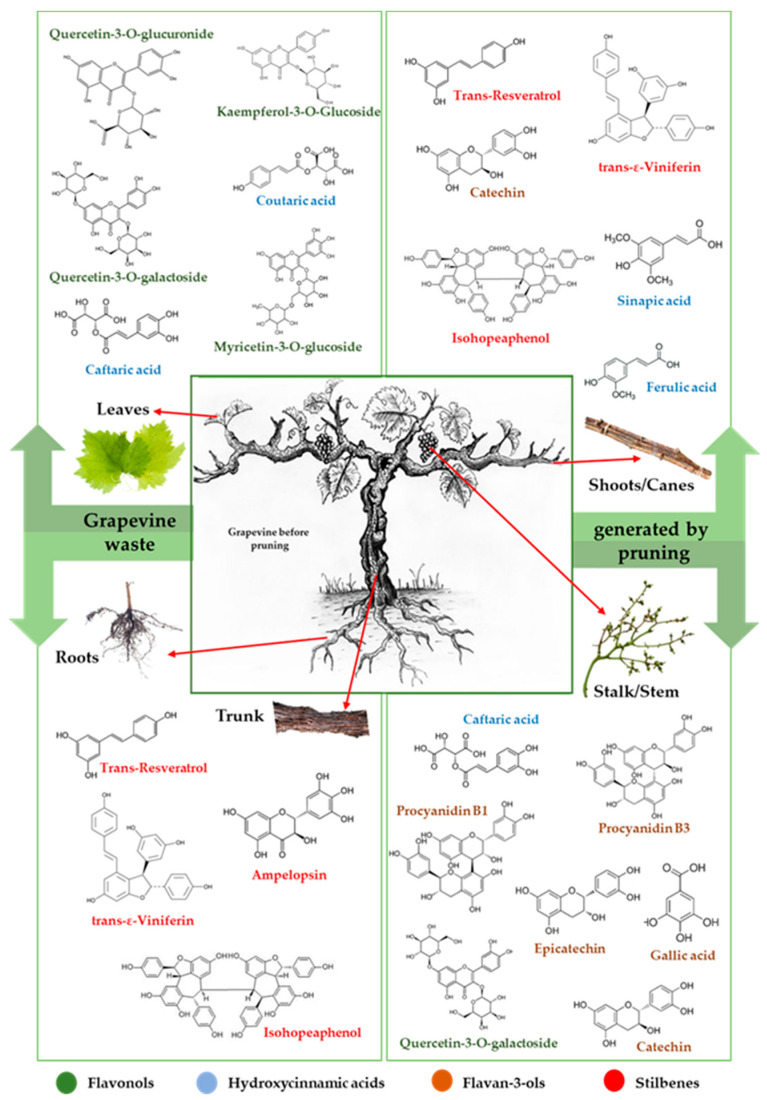
The most abundant polyphenols found in grapevine waste.

Over the years, many extraction methods have been approached in order to recover bioactive compounds with beneficial properties from grapevine wastes. The most abundant polyphenols present in grapevine varieties, according to reviewed literature data, are presented in Figure 3, while some representative results are provided in Table 1.

As can be observed from Table 1, the concentration of active compounds from grapevine wastes strongly depends on several factors, including, but not limited to, the used variety, harvesting time, and extraction method applied. Furthermore, applying a post-harvest treatment in the form of wilting can considerably increase the contents of anthocyanins, proanthocyanidins and stilbenes in the grape composition [33]. Rätsep et al. [27] have conducted a study evaluating the polyphenolic content of grapevines and canes in accordance with the pruning time and dormancy phase of the grapevine. Furthermore, they have shown that the bioactive molecule content of wastes from Hasansky Sladky (hybrid obtained from *Vitis amurensis* L. and *Vitis labrusca* L.), Zilga (a combination of *Vitis amurensis* L. and *Vitis labrusca* L.) and Rondo (*Vitis amurensis* L., used for red wine) can be influenced by hybrid type and the vegetation phase. The microwave extraction method was chosen for this experiment. A solution of 60% ethanol–water (*v/v*) was added to the dried and ground vegetable material. The microwave power used was 100 W for 5 min. In all three cases, unlike the endo-dormancy phase in which the amounts of flavonols increase and those of stilbens decrease, in the eco-dormancy phase increased levels of both flavonols (mainly quercetin-3-glucuronide, quercetin-3-glucoside, quercetin-3-galactoside, and quercetin) and stilbenes (trans-resveratrol and ε-viniferin) were observed.

Prusova et al. [29] have conducted a comparative study between different grape stalks from white (Sauvignon blanc and Grüner Veltliner) and blue (Blauer Portugieser and Cabernet Moravia M-43) grape varieties. The waste material was first crushed and homogenized. The extraction was performed in a dark and cold shaker using 75% methanol as the extraction solvent (the ratio between vegetal material and solvent was 1:9 (*w*/*v*)), with two hours of extraction time. The results show the highest concentrations of syringic acid, caffeic acid, ferulic acid, coumaric acid, trans-resveratrol, catechin and epicatechin in the case of Blauer Portugieser. Sauvignon Blanc presented the highest concentrations of gallic acid and 4-hydroxybenzoic acid, and Cabernet Moravia M-43 contained the highest level of protocatechuic acid. The lowest levels of alpha-amino acids were identified in the Grüner Veltliner variety, and the highest in Blauer Portugieser. The conclusion provided by the authors was that the major differences between the constituent polyphenols from these four stem varieties may present the basis for new research on coniferous fermentation. Subcritical-water extraction, an environmentally friendly technique, was used to extract bioactive compounds with high antioxidant activity from vine canes [28]. Thus, 400 mL of water (used as extraction solvent) was added to 40 g of milled vine canes from Touriga Nacional (TN) and Tinta Roriz (TR) used as raw material. The authors studied the influence of the extraction temperature at 125 °C and 250 °C for 50 min. A total of 1884 mg/100 g d.w. and 1440 mg/100 g d.w. phenolic compounds were obtained for TR and TN, especially phenolic acids, flavanols and stilbenes. The temperature of 250 °C ensures a more efficient extraction, with an increased content of bioactive compounds in both cases of vine canes. The researchers also found a direct dependency between the elevated levels of polyphenols and increased antioxidant activity of the extracts.

In a study conducted by Veskoukis et al. [30], the antioxidant and antimutagenic properties of extracts obtained by ultrasound-assisted extraction from Mavrodaphne, Muscat and Rhoditis stems were demonstrated. For this purpose, 50 g of air-dried and powdered stems was added into 200 mL of mixture of methanol (MeOH)/H_2_O/1.0 N HCl (90:9.5:0.5 *v/v*) as extraction solvent and sonicated for 10 min. The total phenolic content obtained was 374.765 μg/mL, 264.795 μg/mL and 359.865 μg/mL for Mavrodaphne, Muscat, and Rhoditis, respectively. The authors demonstrated that the predominant phenolics in Mavrodaphne are represented by gallic acid, caffeic acid, quercetin and quercitrin, and in Muscat variety by gallocatechin, polydatin and hesperidin. The Rhoditis variety composition was dominated by higher amounts of procyanidin B1 and B2, catechin, epicatechin, dihydroxybenzoic acid, rutin, quercitrin-3-b-glucoside and trans-resveratrol in comparison with the other two varieties. *Salmonella typhimurium* TA102 was used as bacterial strain to determine the antimutagenic capacity of the extracts. The results indicate that the stem extract of Mavrodaphne has a higher antioxidant and antimutagenic capacity than Muscat and Rhoditis (in 2,2-diphenyl-1-picrylhydrazyl-DPPH and superoxide assays).

Caffeic acid, catechin, kaempferol, quercetin and resveratrol were easily extracted from Pinot Noir leaves using suitable metabolic extraction with direct infusion-FT-ICR-MS [34]. Sixteen different types of *Vitis vinifera sativa* canes have been studied for their stilbene content, namely Cabernet franc (CF), Chardonnay (CH), Crimson seedless (CR), Flame seedless (FS), Gewurztraminer (GT), Muscat of Alexandria (MA), Muscat Julius (MJ), Ohanes (OH), Palomino fino (PF), Pinot noir (PN), Regent (RG), Sauvignon blanc (SB), Syrah (SY), Tempranillo (TE), Thomson seedless (TS) and Tin-tilla de Rota (TR). Following the extraction (200 milligrams of powder cane/10 mL of acetone: H_2_O mixture (60:40 *v/v*)) the total stilbene content obtained varied from 2400–5800 mg/kg d.w. Thus, ten stilbenes were identified: hopeaphenol, ampelopsin A, isohopeaphenol, piceatannol, trans-piceid, trans-resveratrol, miyabenol C, ε-viniferin, r-viniferin and ω-viniferin [35]. Alongside the vegetative stage, variety and harvesting period, environmental factors and the properties of the soil can significantly change the chemical composition of leaves [36,37]. Hence, the content of bioactive compounds and antioxidant activity were studied on leaves from eleven different types of vine varieties, grown in the same areas and under the same agronomic conditions by Banjanin et al. [31]. In the composition of the extracts of different leaves, flavonoids are predominant, followed by total phenol, then carotenoid. The major components of vine leaves identified were: 3,4-dihydroxybenzoic acid, (+)-catechin, 1,2-dihydroxybenzene, rutin-trihydrate, quercetin, apigenin-7-glucoside, and caffeic acid. In another study, catechin, epicatechin and procyanidin dimers/trimers (flavan-3-ol class) were identified in high concentrations in the composition of pomace collected from Dunkelfelder 2012, Dunkelfelder 2013, Cabernet Franc, Merlot and Chardonnay [32]. Using pressurized liquid extraction (water as solvent), pomace (vegetal material) and evaluating the optimal extraction temperatures (T = 100 °C/150 °C/200 °C) at P = 25 MPa, flavan-3-ols can be successfully extracted. The highest flavan-3-ols content appeared in the case of Dunkelfelder 2012 (198.86 mg/100 g dry matter/at 200 °C) and decreased in the order Dunkelfelder 2013 > Cabernet Franc > Chardonnay > Merlot (38.70 mg/100 g dry matter/at 100 °C). At 200 °C, (+)-catechin and (-)-epicatechin were most efficiently extracted (from all five types of grape pomace); in contrast, at 150 °C Proanthocyanidins B1 and C1 showed remarkable results in the cases of Cabernet Franc, Merlot and Chardonnay [32].

The presented results support not only the possibilities to recover valuable bioactive compounds from grapevine wastes, but also briefly present the influence of different parameters on their final concentration. All these parameters should be considered when aiming at developing particular tools for targeted applications, based on grapevine wastes.

## 3. Development of Cosmetic Formulations Based on Bioactive Compounds Obtained from Grapevine Wastes

**Figure 4:**
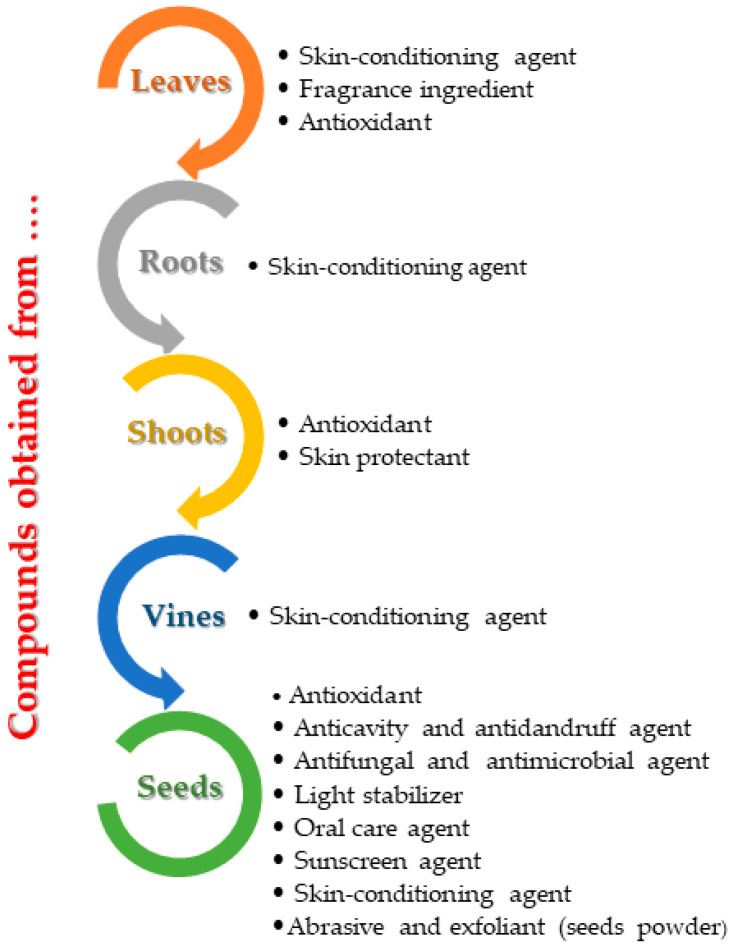
The main applications of bioactive compounds obtained from grapevine waste extracts in cosmetic formulations.

The cosmetics industry includes a wide range of products having as their main role the care, protection and improvement of skin (Figure 4). Given their final application, they can be classified into hygienic (deodorants, soaps), decorative (hair dyes, makeup) and protective (moisturizers, lubricants or sunscreens) products [38]. Each of them contains a basic substance, an active ingredient and a raw material or main ingredient. Classic preservatives in cosmetic formulations, mainly parabens or formaldehyde, exhibit negative effects on an organism, and were eventually replaced by natural ingredients. The process includes the extraction of biologically active principles (especially polyphenols) and their application in cosmetic formulations, as antioxidants for skin care [39]. Various scientific studies confirm the presence of high levels of bioactive compounds in grapevine wastes (especially canes, stems, leaves, etc.), the literature data presenting over 183 phenolic compounds, 78 stilbenes, 15 hydroxycinnamic acids, 9 hydroxybenzoic acids, 17 flavan-3-ols, 14 anthocyanins, 8 flavanoavonols, 2 flavones and 5 coumarins [40]. Many of these biocompounds can be successfully applied for the development of new cosmetic formulations [41].

Polyphenols play an important role in skin functionality, having moisturizing, smoothing, calming, softening and astringent effects. In addition, they soothe irritation and reduce the redness of the skin, accelerate the natural regeneration of the epidermis, and improve the microcirculation and elasticity of the skin [42]. They also protect the skin from harmful external factors, being used as active agents in cosmetic formulations as sun protection ingredients, having the same mechanism of action as chemical UV filters [43]. Oxidative stress, defined by Kawamura et al. [44] as a “disturbance of the oxidation-reduction balance in favor of oxidants”, can eventually lead to damage to biomolecules, changes in metabolism, increased DNA mutations, and an increased rate of cell mitosis [45]. In addition, the impact of oxidative stress on human body can generate inflammatory, cardiovascular, neurodegenerative or metabolic disorders, which in turn can lead to the development of cancer [46]. Free radicals and ROS (reactive oxygen species) are the main oxidizing agents in cellular systems, physiologically produced in various cellular biochemical reactions that occur in the organism, both in mitochondria for aerobic oxygen production, in the metabolism of fatty acids and drugs, and during the activity of the immune system [47]. Furthermore, they are involved in the aging process and in the evolution of many other types of diseases [48]. The aging process can be slowed down by using exogenous and endogenous antioxidants, which can readjust the level of oxidative stress in the human body [49]. The antioxidant effect of polyphenols (including those recovered from vineyard wastes) is to eliminate free radicals O_2_^−^ and OH^−^, by donating a proton from a hydroxyl group attached to the aromatic ring. Thus, they prevent high levels of ROS, reactive nitrogen species and oxidation of sensitive biomolecules, proteins or lipids [46]. Skin aging is a continuous phenomenon, being caused by both internal factors (cellular metabolism, DNA metamorphosis, mitochondrial and genetic dysfunction) [50,51] and external ones (including lifestyle, diet, pollution, smoking, UV light and other environmental factors) [52].

In addition to their antioxidant properties, polyphenols can inhibit the enzymes (tyrosinase, collagenase and elastase) responsible for the aging process of the skin [53,54].

Thus, gallic acid, chlorogenic acid, epicatechin, rutin, and resveratrol, which are found in vine-leaf extract, can inhibit the activity of tyrosinase with an IC_50_ value of 3.84 mg/mL of tyrosinase inhibition, and thus the extracts can be used in cosmetic formulations as a natural whitening agent [55].

In many cases, nanoformulation of resveratrol might be a reliable solution to increase its efficiency because it is unstable against temperature, pH and light and has low solubility in water [56]. Resveratrol-based gel (0.01% weight by volume, applied once a day) may improve the severity of acne and the average surface of microcomedones without any reported side effects [57], while resveratrol-enriched products can ameliorate facial redness [58].

In a recent study, Leal et al. [59] propose the use of grapevine stem extracts (Syrah variety) as raw material in cosmetic products to combat skin wrinkling and pigmentation. In addition, they exhibit anti-inflammatory activity (by inhibiting the nitrite production at non-toxic cell concentrations), anti-aging activity by suppressing the enzymes tyrosinase (53%) and elastase (98.02%), and antimicrobial effects on gram-positive bacteria, having the ability to inhibit the growth of ulcerated bacteria in wounds to the foot [59].

Grape seed oil is known to be rich in unsaturated fatty acids and phenolic compounds [60,61]. Furthermore, high levels of antioxidants from grape seeds exert a protective effect on the skin by increasing cellular resistance and protecting fibroblasts from UV damage by absorbing it [62]. Based on all these considerations, grape seeds can serve as value added to cosmetic formulations [62]. In the composition of some sunscreens, extracts with compounds that exhibit anti-inflammatory activity to reduce UVB-induced erythema or to increase the protection factor (SPF) have been added [63]. In vitro studies on the photostability of the formulation containing 10% *w/w* grape pomace extract and 11.5% *w/w* UV filters showed an SPF value of 16 and an antioxidant activity of 519.92 ± 0.00 μmol Trolox equivalents/g [64]. Furthermore, the methodology proposed by Michailidis et al. [65] proposes the use of grape seed extracts obtained by ultrasound-assisted extraction in dermo-cosmetic products, as anti-elastase and anti-tyrosinase factors [65].

Grape seed extracts (GSE) have been successfully used in the formulation of emulsions and emulgels. In a detailed study, Rafique et al. [66] demonstrated the anti-inflammatory and anti-wrinkle properties of polyphenols from grape seeds, properties that increase skin hydration and elasticity. The proposed emulsion consists of an oily phase containing propylene paraben (preservative)/paraffin oil/Abil-EM 90 (emulsifier)/distilled water/5% grape seed extract. In parallel, the emulgel was formed by mixing the oily phase with an aqueous phase (containing grape seed extract) and finally with a gel phase (created by homogenizing the Carbopol 940 with water). The authors claim that, due to its twice as well-controlled release effect, the emulgel has better anti-aging properties than the emulsion. Another type of oil-in-water emulsion was developed by Yarovaya et al. [62]. In the aqueous phase (containing glycerin and water) xanthan gum was dispersed until a uniform gel was formed. The oily phase was formed in two stages: (i) emulsion consisting of mineral oil, cetyl alcohol and cetamacrogol 1000, over which the aqueous phase and grape seed extract were added and (ii) emulsion containing both grape seed extract and octyl methoxycinnamate. A preservative was added to the mixture of these two parts. The results showed that cells can be protected against UVA radiation if a concentration of 25 μg/mL of GSE is used (this increases the activity of dermal fibroblasts). At the same time, the octyl methoxycinnamate plays an important role in cosmetic formulation because it increases the absorption capacity of the UV filter [62].

Grape cane extracts enriched with polyphenols may activate SIRT1 (a cell longevity protein) and have the ability to inhibit tyrosinase as effectively as pure E-resveratrol and E-ε-viniferin, having utility against dark spots or as skin-lightening agents in eco-dermocosmetic products [67]. In a 28-day study on 60 female subjects, shoot extracts (serum/cream formulation) proved anti-aging effects through increasing radiant glow, evenness, smoothness, hydration, texture, softness effects and decreasing of wrinkles and fine lines [68].

A topical formulation was created by Moreira et al. [69] using subcritical water vine-cane extract with high antioxidant properties. The ingredients used for this purpose were: glycerin (7%)/Carbopol (0.5%)/triethanolamine (0.3%)/preservative (phenoxyethanol/methyl paraben/ethyl paraben/propyl paraben/butyl paraben mixture, 0.1%)/perfume (0.1%). The cosmetic formulation was achieved by dissolving the Carbopol in a mixture of extract/water: 75%/17% ratio at room temperature, while to form a homogeneous gel, triethanolamine was added under continuous stirring. Furthermore, vine cane extracts effectively inhibit the formation of biofilm on *Candida albicans* and *Candida parapsilosis* strains, with a minimum inhibitory concentration (MIC) value of 5 mg/L and 30 mg/L respectively [70].

The extracts obtained from tendrils and leaves of *Vitis vinifera* L. have shown antioxidant activities (in the DPPH and ferric reducing antioxidant power — FRAP assays) and anti-inflammatory capabilities by mitigating the proinflammatory response induced by the exposure to lipopolysaccharides of human gingival fibroblasts cells. The authors suggest that they may be used in oral hygiene products for periodontal disease [71]. In another study, Singla et al. obtained a mouthwash solution based on grape seeds. From in vitro studies, the grape-based oral care formulation showed a reduction of 12.5 % in oral streptococci [72].

In a formulated cream based on an oil/water emulsion, Carica papaya leaf, *Psidium guajava* leaf and *Vitis vinifera* seeds were used as natural preservatives. The obtained emulsion showed a promising antibacterial effect against the proliferation of various microorganisms, as the concentration of grape seed extract was higher [73]. Extracts rich in stilbene, obtained from grapevine cane waste (Ohanes, Regent, Pinot noir and Tin-tilla de Rota), have showed high antioxidant activity. Thus, they can be used as a natural raw material in nutraceutical applications, but also as natural fungicides [35]. Some representative examples regarding the application of compounds from grapevine wastes in cosmetic industry are presented in Table 2.

## 4. Applications in the Food and Beverage Industries

The food industry is one of the main industries that generates different types of waste. Worldwide, the interest in new valorization mechanisms has increased significantly in order to protect resources and the environment [74]. Due to its phytochemical profile, abundant polyphenols and fibers, and exhibiting of high antioxidant and antimicrobial activities, grapevine waste extract (vine shoots, grape stalks and wine lees) might be efficiently used in the food sector as an oenological and functional additive, functional food or even as fillers in food packaging [24]. Therefore, adding grape by-products (pomace) into animals’ diets has been shown to be effective in increasing the nutritional value of their meat. In the same way, they have been added to the diets of poultry, observing the ratio improvement of polyunsaturated and saturated fatty acids [75,76]. Furthermore, the use of grape stems and wine lees grape extracts as feed additives in broilers’ diets improves the quality of the meat [77].

De Iseppi et al. [78] proposed the use of wine yeast glycocompounds (a winemaking by-product) in order to improve both the sensory properties and stability of wine. Results obtained in the case of wine lees extracted by autoclave showed an enhancement of wine foaming along with the efficient recovery of tartrates from its insoluble fraction, and the yeast extracted by enzymatic and ultrasound methods stabilizes the proteins from heat-sensitive wine [78]. Raposo et al. [79] studied extracts from vine shoots, which contain 29% stilbenes, for their potential preservative effect on bottled wine. In the initial phase, the wines treated with shoot extracts presented qualitative superior oenological parameters and higher values of purity and color intensity; unfortunately, these characteristics are not maintained after a year [79]. Additionally, Gutiérrez-Escobar et al. [80] have studied the possibility of the replacement of SO_2_ in wine with pure stilbene extracts from grapevine shoots. The natural extracts, abundant in E-ε-viniferin (70%) and E-resveratrol (18%) and with no aromatic compounds, exhibited high antimicrobial activity against *Brettanomyces bruxellensis* and *Zygosaccharomyces bailli* yeasts strains. Thus, vine shoot extract might be used as a preservative of wine as well as to increase its stilbenes content [80].

Various scientific studies report the applicability of grape stems extracts in the food industry. Phenolic acids, flavanols and tannins from dried and milled vine stems have the ability to remove unstable proteins, being used as a replacement for bentonite (a clay used in wine to avoid protein haze formation) [81]. The hydroalcoholic extracts of grape stems play an important role in the inhibition of food pathogens such as *Listeria monocytogenes*, *Staphylococcus aureus*, *Salmonella enterica* subsp. *enterica* serovar *Typhimurium* and *Escherichia coli* in the cases of lettuce and spinach [82]. By drying, crushing and autolysis of wine yeast, proteins are successfully extracted and applied in the production of fortified cereal bars, improving their protein content [83].

Due to their phytochemical composition, wine lees can also enhance the antioxidant and antimicrobial activity and phenolic compounds in burgers, being used as an alternative to synthetic additives [84]. Phenolic compounds and dietary fiber from wine lees can be also used in the production of high added-value ice cream, conferring better structure, high antioxidant content and inhibitory effect towards the oxidation of human erythrocyte membranes [85], enhancing their physical, chemical and sensory properties, along with protection against *Lactobacillus acidophilus* during storage [86].

Iuga et al. [87] proposed the use of grape seeds and pomace as secondary flours in the production of pasta and pastry products, having the effect of improving the functional ingredients in these branches of the food industry [87]. In the same way, the flours obtained from these types of waste offer physico-chemical characteristics within the nutritional standards, being applied in the biscuit industry [88].

The negative effects of plastic materials on the environment have led the scientific community to develop new biodegradable materials. Thus, insoluble lignocellulosic fibers extracted from grape stalks are used as foams in food packaging, giving them improved mechanical properties, high resistance to moisture and biodegradable characteristics [89]. Díaz-Galindo et al. [90] created a new sustainable food packaging formula based on polylactic acid loaded with grapevine cane extract (5–15 wt%), aiming to prevent food contamination throughout transport and storage. The material showed thermal stability up to 300 °C and resistance values at traction similar to those of commercial materials; the addition of larger amounts of extract increases the breaking strength of the films. Some representative examples regarding the application of compounds from grapevine wastes in food and beverage industry are presented in Table 3.

## 5. Potential Uses of Grapevine Waste-Derived Products in Biomedical Applications

It is well known that the long-term use of commercial synthetic drugs presents side effects on human health [91]. Various scientific papers claim the benefits that polyphenols recovered from different plants in general, and from grapevine wastes in particular, can bring on human health, by protecting the cardiovascular system and neurons as well as anticancer activity [92,93,94,95,96,97]. In different parts of the grapevine, there are different nutritional components such as proteins, lipids, carbohydrates, minerals, vitamins and a wide diversity of bioactive compounds that can have antioxidant, antiviral, antiplatelet, antifungal, anti-cataract, anti-obesity, anticholinergic, and anti-inflammatory effects among others [98].

As mentioned before, winemaking by-products consist of high levels of polyphenols and dietary fiber that fulfil various beneficial roles on human health, namely cardiovascular disease and obesity prevention, control of glucose absorption and the levels of cholesterol in blood [99,100]. One of the main radical generators involved in cell damage is the powerful oxidant called peroxynitrite (ONOO^−^). Thus, quercetin, catechin and epicatechin extracted from grape seeds and skins lead to IC_50_ values of 48.8, 55.7 and 56.7 mM [101].

Two potential inhibitor compounds of amyloid β-protein 25−35 (Aβ) were recovered from grapevine extracts, namely ampelopsin A and piceatannol [102]. It is known that ampelopsin A is responsible for the in vivo protection against brain cell dysfunction by blocking the aggregation of Aβ [103]. In addition, piacetamol (a hydroxyresveratrol) has cardioprotective activity and can also decrease neuronal inflammation in microglial cells [104]. Another compound that can prevent the aggregation of amyloid-β peptides was isolated by Chaher et al. from vine shoot extracts. Thus, the newly isolated compound, Vitisinol C, showed an EC50 value of 5 ± 3 (μmol/L), being proposed for use in the evolution of pharmaceutical therapy for Alzheimer’s disease [105].

Nowadays, it is a generally accepted premise that moderate and regular consumption of red wine might be the key to the prevention of cardiovascular, oncological and neurodegenerative diseases, type 2 diabetes and other chronic diseases [106]. However, winemaking by-products present a much higher total content of anthocyanins, stilbenes, and flavanols, being much more effective in antioxidant therapy than wine itself [107].

Bioactive compounds from wine by-products exert their protective effect on disorders caused by oxidative stress or inflammatory processes [108]. Thus, flavonoids from grape pomace can decrease the production of RONS (reactive oxygen species and nitrogen) by inhibiting the enzymes that produce them, in particular NOX4 (NADPH oxidase 4), eNOS (endothelial nitric oxide synthase), COX2 (ciclooxigenase 2) and SOD1 and 2 (superoxide dismutase 1 and 2), upregulating NF-κB (nuclear factor-kappa B) and downregulating Nrf2 (nuclear factor erythroid 2-related factor 2) pathways [109]. Enzymatic grape pomace extracts can adapt, in vitro, the transcription of 7α-hydroxylase cholesterol and 27-hydroxylase sterol [110], and ex vivo tests in Wistar rats show lowering levels of VLDL cholesterol and triacylglycerol [106]. Ulcerative colitis, induced by acetic acid, showed ulceration, edema and erosions to the colon in laboratory mice. Histological examination presented an improvement in the intensity and distribution of lesions during the treatment with 0.15 and 0.1 mg of grape pomace seeds [111].

Following the evaluation of grape stem extracts, Quero et al. [112] reported the effects they have on cancer cells (Caco-2, MCF-7, and MDA-MB-231) and also on the intestinal barrier (differentiated Caco-2 cells), suggesting them as a promising factor in cancer treatment and in adjustment of ROS in the gastrointestinal tract. The extracts exerted a decreasing effect on the growth of cancer cells, causing death by apoptosis and an inhibitory effect on the antioxidant enzyme TrxR1, which is responsible for the growth of ROS at the cellular level. In the intestinal barrier, bioactive compounds produce an antioxidant effect providing protection to the intestine in the case of disturbances associated with oxidative stress [112]. Similarly, grape seed extracts from the Negramaro variety were found to be able to induce apoptotic cell death in MCF-7 breast cancer cells. Researchers demonstrated that this effect of grape seed extracts is mediated by improving gap-junction-mediated cell–cell communications through reallocating connexin-43 proteins on plasma membranes and controlling cx43 mRNA expression [113]. A preliminary test over 14 days was performed on rats, to which a pretreatment with 4 mL/kg/day grape seed oil (GSO) was applied, following the experimental induction of ischemia by a single administration of isoproterenol (ISO) 45 mg/kg after 14 days. The final results showed that GSO pretreatment has the ability to remarkably decrease the ventricular conduction, the levels of proinflammatory cytokines and the myocardial fraction of creatine kinase, thus providing a cardioprotective effect in ISO-induced myocardial ischemia [114]. In the case of Cyclophosphamide-induced cardiotoxicity (a single dose of 200 mg/kg/b.w.), a pretreatment consisting of grape seed extracts (oral administration on rats, 150 and 300 mg/kg doses for 6 weeks) has the ability to protect the liver and heart tissue, and may also have an ameliorating effect on oxidative and apoptotic biomarkers, as well as the activity of liver and heart function enzymes [115]. Grape seed extracts were also proven to possess the capacity to reduce two digestive enzymes, namely pancreatic lipases and α-glucosidases, thus having utility in preventing obesity [116].

According to Doshi et al. [117], grape seeds and stems may be a new source of insulin secretagogues, suggesting their application in the treatment of type II diabetes. In the presence of these waste extracts, clinical trials on mice showed that, in the pancreatic islets, there is a 2- to 8-fold increase in insulin secretion at a concentration of 5.5 mM and 16.5 mM glucose [117]. One of the major risk factors for cardiovascular disease is represented by hypertension. Thus, Odai et al. [118] conducted a scientific study in which, for 12 weeks, they administered high doses of grape seed proanthocyanidin extract (400 mg) to 6 men and 24 women, all middle-aged and prehypertensive. The final results revealed an improvement in vascular elasticity and a decrease in systolic blood pressure by 13 mmHg after 12 weeks [118].

Two experimental pathways have been adopted by Empl et al. [119] to investigate the possibility of using grapevine shoots extracts as agents in the prevention of human gastrointestinal cancer. In vivo research on ApcMin mice, which were subject to a high-fat diet similar to a human model of adenomatous polyposis, reported that both low and high doses of grapevine shoot extracts have the ability to reduce the number (in males) and volume (in females) of intestinal adenoma. An in vitro experiment was conducted on APC10.1 cells derived from one ApcMin mouse, showing that shoot extracts may reduce the increase in APC10.1 cells by stopping the cycle and cell sequence, as well as by lessening the number of cells [120].

The neuroprotective effects of organic and conventional extracts from grapevine leaves have been analyzed by their ability to diminish protein and lipid damage and by adjustment of enzymatic antioxidant activity. Organic extracts have shown a protective effect on oxidative deterioration (caused by hydrogen peroxide in the brain of rats) of lipids and proteins in the hippocampus and cerebellum tissues. The conventional ones could reduce TBARS (thiobarbituric acid reactive species) levels in the cortex [120]. The MTT test, applied to evaluate the antiproliferative activity of grape leaf extracts on melanoma A375 and SK-MEL cells, revealed that, with increasing water concentrations and methanolic leaf extracts (1.136, 2.27 and 4.54 mg/mL), a decrease in melanoma cell proliferation is observed during 72 h. Thus, the extracts exert an antiproliferative effect comparable to Cisplatinium [121].

Meng et al. [122] induced obesity in mice by applying a high-fat diet, containing 60% kcal from fat. They claim that the intragastric application of leaf extract (400 mg/(kg × day) inhibits the secretion of pancreatic lipase (IC_50_ = 1.18 mg/mL), supports the secretion of fibroblast growth factor-15 (which stops the synthesis of bile acids and fatty acids) and can reduce food intake by suppressing orexigenic neuropeptide-Y. All these aspects can lead to a lower level of serum cholesterol and low-density lipoproteins in triglycerides, while also decreasing the amount of tissue fat. Thus, leaf extracts may be a natural source of components for preventing obesity mediated by neuropeptide-Y and bile acids [122]. Some representative examples regarding the biomedical applications of compounds from grapevine wastes are presented in Table 4.

## 6. Conclusions and Future Perspectives

The winemaking industry represents a major opportunity for obtaining high value by-products with potential industrial applications. As is the case for many other similar wastes, grapevine wastes are a rich source of compounds with antioxidant effects (besides the well-known resveratrol), which can act as potent reactive free radical scavengers or as enzyme activators, as antibacterial, anti-inflammatory, or anti-carcinogenic agents, among other health benefits. Alongside antioxidant compounds, the use of grape biomass is gaining interest as a source of dietary fiber or pigments, for example. In this context, future trends target processes obtaining multiple product based on integrated up-stream and down-stream systems, thus developing sustainable approaches.

From the details given in this review, it could be concluded that grapevine wastes are a rich source of bioactive compounds, which makes them desirable raw materials to scale up extraction processes, conducive to a full and circular exploration of the whole plant. The accomplishment of this goal would lead to superior use of these wastes, in contrast with the current approach, which applies the wastes as fertilizers or feedstock. Advances in research domains may provide further insights into the mechanistic aspects of the health benefits of bioactive compounds obtained from grape waste.

## Figures and Tables

**Table 1:** Bioactive compounds isolated from grapevine wastes ^1^.

Waste Type	Grape Variety	Collecting Period	Extraction Method	Extraction Parameters	Bioactive Compounds Obtained	Ref.
Shoots	Hasansky Sladky (H)	July	Microwave-assisted extraction	Solvent: 60% EtOH-H_2_O solution (*v/v*) (100 mL); 2 g vegetal dried and ground powder material; *M*_w_P = 100 W, t = 5 min;	Flavonols: quercetin-3-glucoside + quercetin-3-galactoside (456.8 ± 51.2 mg/kg d.w.), quercetin-3-glucuronide (4782.8 ± 711.5 mg/kg d.w.), quercetin (98.1 ± 8.9 mg/kg d.w.)	[27]
	Zilga (H)				Flavonols: quercetin-3-glucoside + quercetin-3-galactoside (807.9 ± 46.1 mg/kg d.w.), quercetin-3-glucuronide (4809.4 ± 283.9 mg/kg d.w.), quercetin (420.4 ± 8.0); rutin (193.8 ± 6.6 mg/kg d.w.)	
	Rondo (H)				Flavonols: quercetin-3-glucoside + quercetin-3-galactoside (1201.4 ± 80.1 mg/kg d.w.), quercetin-3-glucuronide (7353.4 ± 579.7 mg/kg d.w.), quercetin (192 ± 13.3 mg/kg d.w.); kaempferol (116.4 ± 5.4 mg/kg d.w.), rutin (517.3 ± 44.0 mg/kg d.w.)	
Canes	Hasansky Sladky (H)	October (EdD)/March (EcD)	Microwave-assisted extraction	Solvent: 60% EtOH-H_2_O solution (*v/v*) (100 mL); 2 g vegetal dried and ground powder material; *M*_w_P = 100 W, t = 5 min;	EdD/EcD: flavanols: (+)-catechin (31.3 ± 1.7/213.7 ± 21.5 mg/kg d.w.); (-)-epicatechin (2.7 ± 0.2/37.2 ± 1.8 mg/kg d.w.); procianidin B (1.3 ± 0.3/32.4 ± 3.2 mg/kg d.w.); Stilbenes: ε-viniferin (765.6 ± 87.4/1042.8 ± 24.2 mg/kg d.w.); resveratrol (32.4 ± 7.9/186.9 ± 3.4 mg/kg d.w.)	[27]
	Zilga (H)				EdD/EcD: flavanols: (+)-catechin (36.0 ± 5.3/224.5 ± 35.3 mg/kg d.w.); (-)-epicatechin (6.1 ± 1.4/61.8 ± 7.2 mg/kg d.w.); procianidin B (3.2 ± 1.4/49.2 ± 8.6 mg/kg d.w.); Stilbenes: ε-viniferin (595.5 ± 69.6/931.6 ± 72.8 mg/kg d.w.); resveratrol (21.4 ± 2.9/44.0 ± 2.4 mg/kg d.w.)	
	Touriga Nacional	November	Subcritical-water extraction	Solvent: H_2_O (400 mL); 40 g of milled vine-canes; T_1_ = 125 °C/T_2_ = 250 °C; t = 50 min;	T_1_/T_2_: Phenolic acids: gallic acid (60.1 ± 3.0/891 ± 45 mg/100 g d.w.), protocatechuic acid (33.8 ± 1.7/14.5 ± 0.7 mg/100 g d.w.), 4-hydroxyphenilacetic acid (16.8 ± 0.8/62.6 ± 3.1 mg/100 g d.w.), 4-hydroxybenzoic acid (9.2 ± 0.5/22.6 ± 1.1 mg/100 g d.w.), vanillic acid (15.0 ± 0.7/15.6 ± 0.8 mg/100 g d.w.); flavanols: (+)-catechin (102 ± 5/181 ± 9 mg/100 g d.w.); stilbenes: resveratrol (8.0 ± 0.4/15.8 ± 0.8 mg/100 g d.w.)	[28]
	Tinta Roriz				T_1_/T_2_: Gallic acid (74.2 ± 3.7/1066 ± 53 mg/100 g d.w.), protocatechuic acid (33.4 ± 1.7/21.2 ± 1.1 mg/100 g d.w.), 4-hydroxyphenilacetic acid (49.2 ± 2.5/134 ± 7 mg/100 g d.w.), 4-hydroxybenzoic acid (8.4 ± 0.4/44.9 ± 2.2 mg/100 g d.w.), Vanillic acid (13.5 ± 0.7/31.6 ± 1.6 mg/100 g d.w.); Flavanols: (+)-catechin (245 ± 12/216 ± 11 mg/100 g d.w.); Stilbenes: resveratrol (10.5 ± 0.5 13.1 ± 0.7 mg/100 g d.w.);	
Stems	Sauvignon blanc	Late September	Solid-liquid extraction (shaker)	Solvent: 75% MeOH (90 mL); 10 g vegetal crushed material; t = 2 h in a dark and cold basic shaker;	Hydroxybenzoic acid: gallic acid (4.015 mg/L), 4-hydroxybenzoic acid (0.076 mg/L), syringic acid (0.349 mg/L)	[29]
	Blauer Portugieser	October			Gallic acid (0.822 mg/L), Hydroxybenzoic acid: syringic acid (1.346 mg/L), Hydroxycinnamic acid: caffeic acid (20 mg/L) ferulic acid (n.p.), coumaric acid (n.p.); stilbenes: trans-resveratrol (n.p.), Flavan-3-ol: epicatechin and catechin (n.p.)	
	Cabernet Moravia M-43	November			Hydroxybenzoic acid: protocatechuic acid (1.201 mg/L)	
	Mavrodaphne	n.p.	Ultrasound-assisted extraction	Solvent: mixture of MeOH/H_2_O/1.0 N HCI = 90:9.5:0.5 *v/v* (200 mL); 50 g of air-dried and powder stems; t = 10 min;	gallic acid (5.581 μg/mg extract), caffeic acid (3.700 μg/mg extract), quercetin (0.620 μg/mg extract), quercitrin (0.152 μg/mg extract)	[30]
	Muscat				Gallocatechin (0.089 μg/mg extract), polydatin (0.131 μg/mg extract), hesperidin (0.058 μg/mg extract)	
	Rhoditis				procyanidin B1/B2 (10.010/2.999 μg/mg extract), catechin (3.602 μg/mg extract), epicatechin (1.678 μg/mg extract), 2,5-dihydroxybenzoic acid (0.332 μg/mg extract), rutin (0.287 μg/mg extract), quercitrin-3-b-glucoside (0.761 μg/mg extract), trans-resveratrol (0.469 μg/mg extract)	
Leaves	Zilavka	May	Classical water extraction (water bath)	Solvent: MeOH:H_2_O (70:30 *v/v*) (40 mL) + HCl (0.1%); 2 g of dried and grounded leaves; t = 60 min.	3,4-dihydroxybenzoic acid (116.08 ± 2.56 mg/100 g), (+)-catechin (174.38 ± 5.79 mg/100 g), 1,2-dihydroxybenzene (57.82 ± 2.50 mg/100 g), rutin-trihydrate (117.20 ± 3.35 mg/100 g), quercetin (30.77 ± 0.66 mg/100 g), apigenin-7-glucoside (14.75 ± 0.94 mg/100 g), caffeic acid (35.14 ± 0.53 mg/100 g)	[31]
	Royal				3,4-dihydroxybenzoic acid (84.52 ± 2.34 mg/100 g), (+)-catechin (212.46 ± 0.38 mg/100 g), 1,2-dihydroxybenzene (134.13 ± 2.64 mg/100 g), rutin-trihydrate (70.17 ± 1.15 mg/100 g), quercetin (57.09 ± 4.33 mg/100 g), apigenin-7-glucoside (45.58 ± 0.02 mg/100 g), caffeic acid (50.05 ± 1.54 mg/100 g); resveratrol (73.78 ± 1.34 mg/100 g)	
	Merlot				3,4-dihydroxybenzoic acid (82.90 ± 5.47 mg/100 g), (+)-catechin (130.51 ± 3.23 mg/100 g), 1,2-dihydroxybenzene (88.21 ± 2.61 mg/100 g), rutin-trihydrate (32.67 ± 0.42 mg/100 g), quercetin (20.78 ± 0.30 mg/100 g), apigenin-7-glucoside (20.84 ± 0.52 mg/100 g), caffeic acid (32.19 ± 0.98 mg/100 g)	
	Cardinal				3,4-dihydroxybenzoic acid (12.68 ± 0.82 mg/100 g), (+)-catechin (99.99 ± 3.23 mg/100 g), 1,2-dihydroxybenzene (122.69 ± 1.98 mg/100 g), rutin-trihydrate (17.66 ± 1.52 mg/100 g), quercetin (18.09 ± 0.90 mg/100 g), apigenin-7-glucoside (12.70 ± 1.00 mg/100 g), caffeic acid (27.89 ± 0.29 mg/100 g)	
	Blatina				3,4-dihydroxybenzoic acid (93.07 ± 2.59 mg/100 g), (+)-catechin (208.00 ± 0.40 mg/100 g), 1,2-dihydroxybenzene (196.08 ± 4.56 mg/100 g), rutin-trihydrate (39.96 ± 0.21 mg/100 g), quercetin (12.73 ± 1.24 mg/100 g), apigenin-7-glucoside (29.88 ± 0.7 mg/100 g), caffeic acid (28.23 ± 0.54 mg/100 g)	
	Trnjak				3,4-dihydroxybenzoic acid (54.75 ± 0.60 mg/100 g), (+)-catechin (31.85 ± 0.07 mg/100 g), 1,2-dihydroxybenzene (102.90 ± 3.08 mg/100 g), rutin-trihydrate (27.69 ± 0.33 mg/100 g), quercetin (46.23 ± 0.15 mg/100 g), apigenin-7-glucoside (222.49 ± 0.76 mg/100 g), caffeic acid (22.15 ± 0.54 mg/100 g)	
	Sugraone Seedless				3,4-dihydroxybenzoic acid (77.05 ± 0.10 mg/100 g), (+)-catechin (110.65 ± 2.90 mg/100 g), 1,2-dihydroxybenzene (57.56 ± 0.93 mg/100 g), rutin-trihydrate (33.27 ± 0.15 mg/100 g), quercetin (37.90 ± 0.29 mg/100 g), apigenin-7-glucoside (35.71 ± 3.72 mg/100 g), caffeic acid (142.55 ± 0.53 mg/100 g)	
	Cabernet Sauvignon				3,4-dihydroxybenzoic acid (74.01 ± 2.27 mg/100 g), (+)-catechin (119.28 ± 1.28 mg/100 g), 1,2-dihydroxybenzene (69.29 ± 1.71 mg/100 g), rutin-trihydrate (93.21 ± 0.40 mg/100 g), quercetin (112.67 ± 0.74 mg/100 g), apigenin-7-glucoside (22.68 ± 0.02 mg/100 g), caffeic acid (50.57 ± 1.12 mg/100 g)	
	Chardonnay				3,4-dihydroxybenzoic acid (85.33 ± 1.21 mg/100 g), (+)-catechin (133.19 ± 1.66 mg/100 g), 1,2-dihydroxybenzene (160.00 ± 4.23 mg/100 g), rutin-trihydrate (55.23 ± 0.01 mg/100 g), quercetin (19.04 ± 1.11 mg/100 g), apigenin-7-glucoside (95.23 ± 0.34 mg/100 g), caffeic acid (152.27 ± 0.25 mg/100 g)	
	Viktorija				3,4-dihydroxybenzoic acid (88.85 ± 0.78 mg/100 g), (+)-catechin (103.50 ± 2.16 mg/100 g), 1,2-dihydroxybenzene (195.07 ± 0.74 mg/100 g), rutin-trihydrate (57.84 ± 0.30 mg/100 g), quercetin (30.38 ± 2.49 mg/100 g), apigenin-7-glucoside (100.39 ± 1.50 mg/100 g), caffeic acid (47.15 ± 0.15 mg/100 g)	
	Vranac				3,4-dihydroxybenzoic acid (158.97 ± 0.96 mg/100 g), (+)-catechin (158.64 ± 0.54 mg/100 g), 1,2-dihydroxybenzene (270.92 ± 1.51 mg/100 g), rutin-trihydrate (79.24 ± 1.53 mg/100 g), quercetin (32.27 ± 0.73 mg/100 g), apigenin-7-glucoside (222.49 ± 0.76 mg/100 g), caffeic acid (55.73 ± 1.11 mg/100 g)	
Pomace	Dunkelfelder 2012	Provided during 2012	Pressurized liquid extraction	Solvent: H_2_O; 100 g grape pomace; T_1_ = 100 °C/T_2_ = 150 °C/T_3_ = 200 °C; P = 25 MPa;	T_1_/T_2_/T_3_: Catechin (25.62 ± 1.95/35.75 ± 1.09/65.84 ± 2.74 mg/100 g d.w.), epicatechin (18.83 ± 0.44/18.65 ± 0.31/28.49 ± 0.70 mg/100 g d.w.), procyanidin dimers/trimers (4.96± 0.77/18.65 ± 0.31/9.75 ± 0.31 mg/100 g d.w.)	[32]
	Dunkelfelder 2013	Provided during 2013			T_1_/T_2_/T_3_: Catechin (19.49 ± 2.30/31.14 ± 1.13/59.37 ± 2.79 mg/100 g d.w.), epicatechin (13.12 ± 0.53/17.55 ± 1.21/24.69 ± 1.24 mg/100 g d.w.), procyanidin dimers/trimers (4.27 ± 0.39/6.65 ± 0.79/7.23 ± 0.68 mg/100 g d.w.)	
	Cabernet Franc				T_1_/T_2_/T_3_: Catechin (15.17 ± 1.01/18.29 ± 1.50/21.29 ± 0.32 mg/100 g d.w.), epicatechin (13.87 ± 1.06/15.13 0.47/17.54 ± 0.90 mg/100 g d.w.), procyanidin dimers/trimers (2.91 ± 0.17/2.89 ± 0.64/2.29 ± 0.40 mg/100 g d.w.)	
	Merlot				T_1_/T_2_/T_3_: Catechin (7.32 ± 11.65/11.65 ± 0.67/15.48 ± 0.74 mg/100 g d.w.), epicatechin (5.20 ± 6.86/6.86 ± 1.09/3.03 ± 1.56), procyanidin dimers/trimers (2.08 ± 0.18/2.82 ± 0.33/0.49 ± 0.35 mg/100 g d.w.)	
	Chardonnay				T_1_/T_2_/T_3_: Catechin (26.13 ± 2.40/28.48 ± 1.08/31.91 ± 0.97 mg/100 g d.w.), epicatechin (8.24 ± 0.80/12.03 ± 0.17/14.53 ± 0.48 mg/100 g d.w.), procyanidin dimers/trimers (5.58 ± 0.08/6.04 ± 0.46/5.88 ± 0.22 mg/100 g d.w.)	

^1^ where: d.w. = dry weight; EdD: Endo-dormancy; EcD = Eco-Dormancy; EtOH = ethanol; H = hybrid; H_2_O = water; HCl = chlorohydric acid; MeOH = methanol; *M*_w_P = microwave power; n.p. = not provided by the authors; P = pressure; t = time; T = temperature.

**Table 2:** Some examples of grapevine wastes application in cosmetic formulations.

Extracted Wastes	Formulation	Potential Application	Ref.
Vine canes	Topical formulation: vine-cane extract/glycerin (7%)/carbopol (0.5%)/triethanolamine (0.3%)/preservative (phenoxyethanol/methyl paraben/ethyl paraben/propyl paraben/butyl paraben mixture, 0.1%)/perfume (0.1%).	Protection against different oxidants	[69]
	Extracts enriched with polyphenols	Utility against dark spots or as skin-lightening agents	[67]
Vine shoots	Serum: vine shoot extract 0.045%/biotechnological extract—Ronacare Hydroine 1%	Anti-aging effects	[68]
Grape seeds	Emulsion: oily phase containing propylene paraben (preservative)/paraffin oil/Abil-EM 90 (emulsifier)/distilled water/5% grape seed extract	Anti-aging	[66]
	Emulgel: oily phase + aqueous phase (containing grape seeds extract) + gel phase (Carbopol 940/water)	Anti-aging	
	Emulsion: 5% mineral oil/7% cetomacrogol 1000/2% cetyl alcohol/7% octyl methoxycinnamate/3% grape seed extract/1% xanthan/5% glycerin/0.5% phenoxyethanol/purified water qs to 100	UVA protection	[62]
	Extract obtained via an ultrasound-assisted method	Anti-elastase and anti-tyrosinase factors in dermo-cosmetics	[65]
Grape pomace	Sunscreen: 10% *w/w* grape pomace extract and 11.5% *w/w* UV (Butylmethoxydibenzoyl methane—UVA, ethylhexyl methoxycinnamate and ethylhexyl dimethyl PABA-UVB)	UV protection	[64]
	Extract as raw material	Combat skin wrinkling and pigmentation/ability to inhibits the growth of ulcerated bacteria in wounds to the foot	[59]

**Table 3:** Applications of grapevine wastes-derived products in food and beverage industry.

Type of Wastes	Application	Effect	Ref.
Grape pomace	Added into animals’ diets	Increasing the nutritional value of meat	[75]
	Pasta and pastry products as secondary flours	Growing functional ingredients in food industry	[87]
Grape stems/stalks	Disinfectants in cases of leafy fresh vegetables: lettuce and spinach	Inhibition of pathogens *Listeria monocytogenes*, *Staphylococcus aureus*, *Salmonella enterica* subsp. *enterica* serovar Typhimurium and *Escherichia coli*	[82]
	Substitute for bentonite in wine	Removing unstable proteins	[81]
	Food packaging as foams	Increased mechanical properties, high resistance to moisture, biodegradable characteristics	[89]
Grapevine canes	Food packaging formula (polylactic acid loaded with grapevine cane ex-tract)	Prevent food contamination during transport and storage; increases the breaking strength of the packaging films	[90]
Grape stems and wine lees	Feed additives in broilers’ diets	Improvement of meat quality	[77]
Vine shoots	Preservative of wine	Increased quality of oenological parameters and higher values of purity and color intensity	[79]
	Replacement of SO_2_ in wine	High antimicrobial activity against *Brettanomyces bruxellensis* and *Zygosaccharomyces bailli*; increased wine stilbene content	[80]
Wine lees	Wine industry	Reversing wine foam and stabilizing proteins in heat-sensitive wine	[78]
	Development of fortified cereal bars	Improving protein content	[83]
	Alternative to synthetic additives	Enhancement of antioxidant and antimicrobial activity in burgers	[84]
	Production of high added-value ice cream	Superior structure, high antioxidant effect, oxidation inhibition on human erythrocyte membranes	[85]
	Production of high added-value ice cream	Enhanced physical, chemical and sensory properties, protection against *Lactobacillus acidophilus* during storage	[86]

**Table 4:** Examples of biomedical applications of compounds from grapevine wastes.

Waste	Type of Study	Biomedical Activity	Effect	Ref.
Grape leaves	In vivo	Antiproliferative	Reduce melanoma A375 and SK-MEL cells proliferation over 72 h; induce antiproliferative effect comparable to Cisplatinium	[121]
		Neuroprotective	Protection against oxidative deterioration of lipids and proteins in the hippocampus and cerebellum tissues; reduce levels of thiobarbituric acid reactive species in the cortex	[120]
		Obesity prevention	Inhibit the secretion of pancreatic lipase; increase the secretion of fibroblast growth factor-15; decrease levels of serum cholesterol and low-density lipoproteins in triglycerides; reduced the amount of tissue fat	[122]
Grape seeds			Decrease pancreatic lipases and α-glucosidases	[116]
Grape pomace	In vitro	Anti-cholesterol	Transcription of 7α-hydroxylase cholesterol and 27-hydroxylase sterol	[110]
	Ex-vivo		Reduce VLDL cholesterol and triacylglycerol	[106]
	In vivo	Inflammatory bowel disorders prevention	Decrease intensity and distribution of ulcerations, edema and erosions in the colon	[111]
Grape shoots	In vivo	Anticarcinogenic	Decrease the number of intestinal adenoma (male mice); decrease the volume of intestinal adenoma (female mice)	[119]
	In vitro		Reduce the increasing of APC10.1 cells number; stopping the cycle and cell sequence	
Grape stems	In vivo		Reduce growth of Caco-2, MCF-7, and MDA-MB-231 cancer cells; inhibition effect on the enzyme TrxR1; protection of the intestine	[112]
Grape seeds			Induce apoptotic cell death to MCF-7 cancer cells	[113]
		Cardioprotective	Reduce ventricular conduction; decrease levels of proinflammatory cytokines; reduce myocardial fraction of creatine kinase; protective effect in ISO-induced myocardial ischemia	[114]
			Ameliorating effect on oxidative and apoptotic biomarkers; ameliorating activity of liver and heart function enzymes	[115]
		Hypertension prevention	Improvement of vascular elasticity; reduced systolic blood pressure by 13 mmHg after 12 weeks	[118]
Grape stems and seeds		Type II diabetes prevention	Increased insulin secretion in the pancreatic islets	[117]
